# Characterizing viral samples using machine learning for Raman and absorption spectroscopy

**DOI:** 10.1002/mbo3.1336

**Published:** 2022-12-05

**Authors:** Miad Boodaghidizaji, Shreya Milind Athalye, Sukirt Thakur, Ehsan Esmaili, Mohit S. Verma, Arezoo M. Ardekani

**Affiliations:** ^1^ School of Mechanical Engineering Purdue University West Lafayette Indiana USA; ^2^ Department of Agricultural and Biological Engineering Purdue University West Lafayette Indiana USA; ^3^ Weldon School of Biomedical Engineering Purdue University West Lafayette Indiana USA; ^4^ Birck Nanotechnology Center Purdue University West Lafayette Indiana USA

**Keywords:** absorption spectroscopy, convolutional neural network, principal component analysis, Raman spectroscopy, random forest

## Abstract

Machine learning methods can be used as robust techniques to provide invaluable information for analyzing biological samples in pharmaceutical industries, such as predicting the concentration of viral particles of interest in biological samples. Here, we utilized both convolutional neural networks (CNNs) and random forests (RFs) to predict the concentration of the samples containing measles, mumps, rubella, and varicella‐zoster viruses (ProQuad®) based on Raman and absorption spectroscopy. We prepared Raman and absorption spectra data sets with known concentration values, then used the Raman and absorption signals individually and together to train RFs and CNNs. We demonstrated that both RFs and CNNs can make predictions with *R* ^2^ values as high as 95%. We proposed two different networks to jointly use the Raman and absorption spectra, where our results demonstrated that concatenating the Raman and absorption data increases the prediction accuracy compared to using either Raman or absorption spectrum alone. Additionally, we further verified the advantage of using joint Raman‐absorption with principal component analysis. Furthermore, our method can be extended to characterize properties other than concentration, such as the type of viral particles.

## Graphical abstract

We applied machine learning techniques to Raman and absorption spectra to determine the concentration of samples containing viral particles (measles, mumps, rubella, and varicella‐zoster viruses). We proposed two different networks to jointly use the Raman and absorption spectra, where our results demonstrated that concatenating the Raman and absorption data increases the prediction accuracy compared to using either Raman or absorption spectrum alone. Ultimately we were able to make predictions with accuracies as high as 95%.

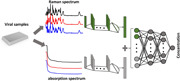

## 1 INTRODUCTION

The recent outbreak of COVID‐19 proved the importance of robust antiviral medications to stop the spread of pandemic viral infections. Antiviral drugs and vaccines are the two major solutions to keep viral infections at bay. A recent study suggested that, for example, the COVID‐19 vaccine saved approximately 20 million human lives in 1 year (Watson et al., 2022). Measles, mumps, rubella, and varicella (MMRV) are common viral childhood diseases that can have serious complications. Developing efficient methods to mass produce MMRV paves the way for limiting the spread of the MMRV globally. The vaccine development flourished in the early 20th century, and Maurice Hilleman at Merck & Co Inc., a pioneer in the development of vaccinations, developed Rubeovax™ in 1968, the first commercial live vaccine for measles (Tulchinsky, 2018). Vaccine development and production have been continuously improving in upstream and downstream processing (Blue et al., 2015). Vaccine production involves challenging processes such as viral vector development, effective purification, polishing steps, and formulation with stable storage conditions. These processes require comprehensive and continuous quality management to maintain the product's efficacy and ensure public safety. With the advancement in viral vector‐driven gene therapies and vaccine production, there is a growing interest in improving the continuous production of virus‐like particle (VLP)‐based vaccines (Gutierrez‐Granados et al., 2018). The development of continuous manufacturing processes in the vaccine industry demands rapid, robust, and continuous analytical methods (Process analytical technology [PAT] tools) to understand real‐time manufacturing processes (Maruthamuthu, Rudge, et al., 2020).

Noninvasive in‐line sensors such as Raman probes (Raman spectroscopy) hold great potential due to their higher sensitivity to read the molecular fingerprints of chemical and biological molecules, species, or products (Butler et al., 2016; Rolinger et al., 2020). Raman spectra possess clear spectral features that can be easily assigned to different chemical compounds. Additionally, minimal sample preparation is sufficient for making accurate quantitative predictions using Raman spectra (Pian et al., 2022). In other words, Raman spectroscopy provides invaluable information for various analyte molecules even in ultra‐low concentrations (Panneerselvam et al., 2022). Similarly, absorption spectroscopy is a robust technique that, owing to its high sensitivity and large signal‐to‐noise ratio, (Torrisi et al., 2020) has the potential to be implemented as a great tool to make predictions. Generally, both Raman and absorption spectra have been widely used for particle detection and identification (Barnes et al., 2006; Nitkowski et al., 2008; Pallaoro et al., 2015; Probst et al., 2021) and quantitative analysis (Bao et al., 2018; Storey & Helmy, 2019; Strachan et al., 2007).

Recently machine learning (ML) has become popular for making predictions based on spectroscopy data. Both supervised and unsupervised ML techniques have been applied to Raman signals to make predictions (Ralbovsky & Lednev, 2020). Particularly, Raman spectroscopy has been utilized for cancer predictions (Ralbovsky & Lednev, 2020). For instance, techniques, such as principal component analysis or artificial neural networks have been utilized for detecting cervical cancer (Daniel et al., 2018). Furthermore, Raman signals have been utilized for classification problems, such as classifying bacteria (Khan et al., 2018; Koya et al., 2018; Maruthamuthu, Raffiee, et al., 2020; Maruthamuthu, Rudge, et al., 2020) viral, (Ditta et al., 2019; Tong et al., 2019) and fungal infections (Dzurendová et al., 2021; Guo et al., 2021). Additionally, Raman spectroscopy has been applied for regression purposes, such as predicting the concentration of the markers of interest, such as sensing the pH and Lactate in body fluids (Olaetxea et al., 2020). Absorption spectroscopy also has been utilized for classification purposes, such as the characterization of proteins (Zhang et al., 2021) classification of wines, (Philippidis et al., 2020) and quantifying the concentration of organic acids (Wolf et al., 2013). Furthermore, the joint Raman and absorption spectra have been applied to predict the values of concentrations (Isaev et al., 2020).

Previous studies, in particular, have confirmed the capability of ML techniques in making quantitative predictions based on Raman or absorption signals. However, a comparison of these signals and their strength in making accurate ML‐based predictions for viral samples, such as MMRV has not been studied before. Here, we aim to create methods based on Raman and absorption spectroscopy that enables monitoring of the concentration of the viral particles in well plates. Additionally, it is not known whether using Raman and absorption spectra simultaneously can boost the prediction accuracy compared to using only Raman or absorption spectra separately. In our previous study, we demonstrated that deep learning enables the efficient detection of bacteria, fungi, and mammalian cells in static dried‐down conditions (Maruthamuthu, Raffiee, et al., 2020). Following our previous study, we intend to build convolutional neural networks (CNNs) and random forests (RFs) models that accept the Raman or absorption spectra or their combination as the input and predict the concentration of samples containing MMRV.

## 2 MATERIALS AND METHODS

### 2.1 Data acquisition

All these samples prepared in this study are based on the ProQuad®, which is a sterile, lyophilized, preservative‐free, live virus vaccine that contains measles, mumps, rubella, and varicella‐zoster viruses (Kuter et al., 2006). We procured ProQuad® (manufactured by Merck & Co Inc.,) from the Purdue College of pharmacy and stored it at −20°C. We prepared the linear dilutions of the ProQuad® vaccine with a step size of 4% and an initial concentration of 7.20E + 05 plaque‐forming units/ml (PFU/ml) (Lyophilized ProQuad® + 10 µl Diluent). Throughout this article, we refer to the number of infective particles within the sample (PFU) as particles. All the Raman spectra of the ProQuad® dilutions were collected with the Renishaw in Via^TM^Qontorconfocal Raman microscope (Renishaw plc) (RENISHAW). We used a 785‐nm excitation laser with 100% (300 mW) power and 10 s acquisition time (1 accumulation). The spectral resolution of the spectra was 1 cm^−1^, and the spectrum ranged from 101 to 3200 cm^−1^ corresponding to 3194 Raman shifts. The samples were focused with an X5 objective of a microscope (LeicaDM2700M), and three replicate Raman spectra were collected for each dilution. The sample volume used for the measurement was 100 µl, and the substrate used for the measurements was a 96‐well plate (Corning^TM^3635 UV‐Transparent Microplates). The experiment was repeated once. The raw Raman spectral data was collected using WiRE 5.5 software. Furthermore, we collected the absorption spectrum for ProQuad® dilutions using the BMG LABTECH, Inc microplate reader (CLARIOstar Plus, SN: 430‐2173). The spectrum range was 220 to 1000 nm with a spectral resolution of 1 nm wavelength corresponding to 781 wavelengths. The sample volume used for the measurement was 100 µl, and the substrate used for the measurements was a 96‐well plate (Corning^TM^3635 UV‐Transparent Microplates). We collected three spectral scans for each dilution. The experiment was repeated once. In total, the data set includes Raman and absorption spectra for 25 different concentration values with 3 to 6 replicates for each value, making a total of 116 samples, where 20% of this data is used for testing by 5‐fold cross‐validation as described in Section 2.2.

### 2.2 Machine learning modeling

We adopt two widely used ML techniques to relate the Raman and absorption spectra to the concentration values: the RF and the CNN techniques. Before training, to ensure the reproducibility of the results, all the models are initialized by setting the seed number to zero. To assess the accuracy of predictions, we use the values of the coefficient of determination (*R* ^2^ scores). Further, to train the models, the 5‐fold cross‐validation technique is used both for the CNNs and RFs. In this method, the whole data is split into five sections, where the model is trained five times, and each time four sections are used as the training data set and one section as the testing data set. The 5‐fold cross‐validation model ensures that all the data points fall into the testing data set at least once, preventing biased predictions. The Sklearn (Pedregosa et al., 2011) and Pytorch (Paszke et al., 2019) modules in Python are used for modeling the RFs and CNNs, respectively.

**Figure 1:**
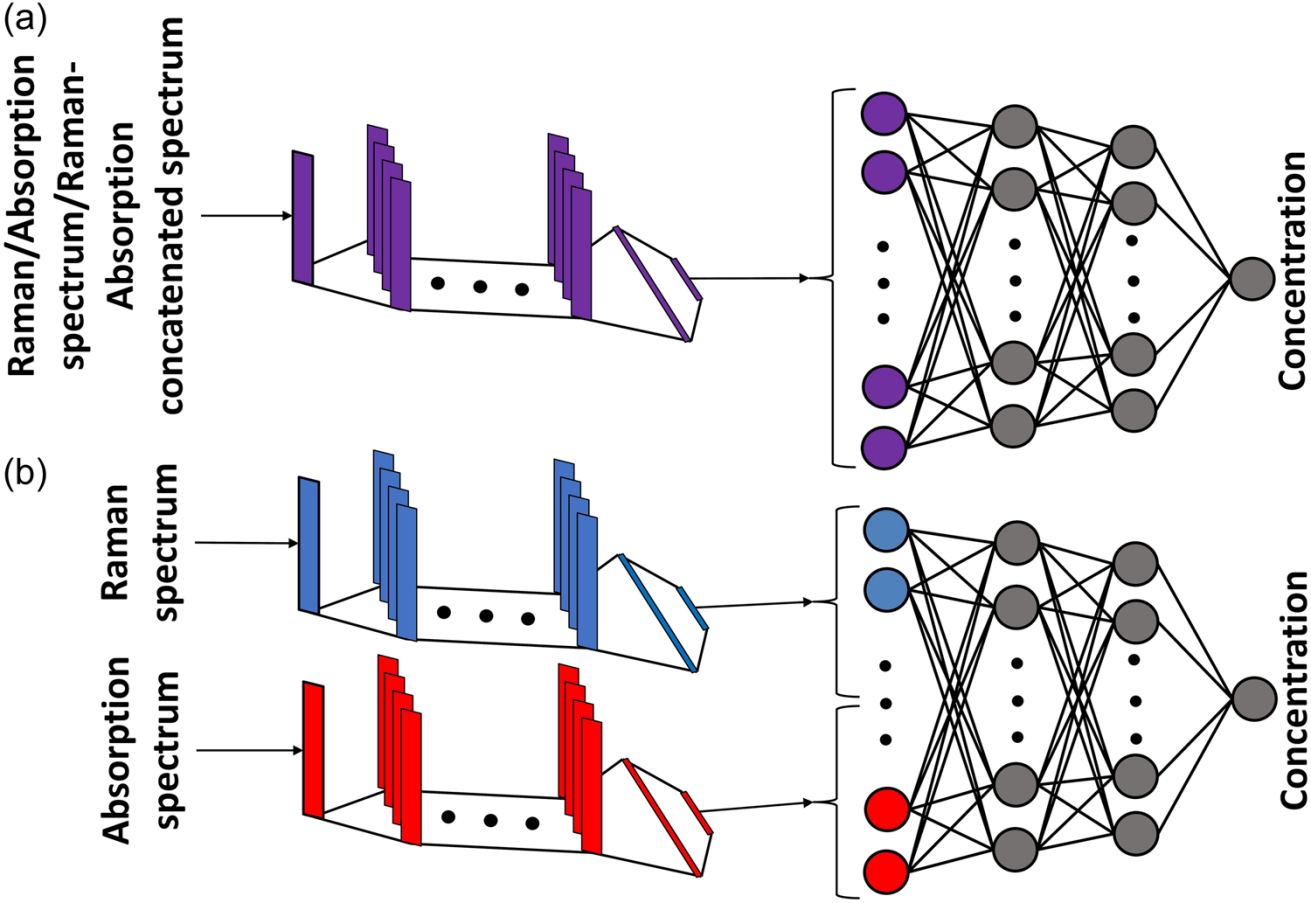
Schematic view of the neural network structure when (a) Raman, absorption, or concatenated Raman‐absorption spectrum is used as the input (b) both Raman and absorption spectra are used as separate inputs. The number of layers shown here is for illustration purpose and does not reflect the actual values.

CNN is a supervised machine learning technique that, in our case, takes one‐dimensional signals as the input and identifies the important parts of the signal, which paves the way for automatic learning of various features and hidden aspects in the signal that are important for the regression. In other words, CNN can capture the spatial and temporal dependencies in the Raman or absorption spectrum. The general architectures of the deep learning models used in this study are similar, that is, a feed‐forward single CNN consisting of four convolutional layers followed by four fully connected layers when either Raman or absorption spectrum is used as the input, as shown in Figure 1a. However, when it comes to using both the Raman and absorption spectra as the input, we use two different designs. In one design, we concatenate the Raman and absorption signals and feed them into a single CNN, as shown in Figure 1a. In another design, a double CNN is created for feeding the inputs, as demonstrated in Figure 1b. In the double CNN, the Raman and absorption spectrum are first fed into two separate networks with four convolutional layers and then two fully connected layers. Eventually, the outputs of each network are concatenated and fed into a network with two fully connected layers. In all models, the architecture used for convolutional layers is based on residual mapping following the deep residual learning method (He et al., 2016). The presence of residual blocks with shortcut connections between inputs and outputs boosts the training stability and paves the way for having deeper layers (He et al., 2016).

Furthermore, the kernel size used for all the convolutional layers is three with zero paddings and strides of one. Additionally, all the networks are trained for 6000 epochs (iterations), where a further increase in the epochs does not significantly boost the prediction accuracy. We use the mean squared loss function as the criterion for training with the back‐propagation techniques, where we adopt the stochastic gradient descent with momentum and adaptive learning rate, Adam, (Kingma & Ba, 2014) where the weight decay and learning rate are set to 0.1 and 10^−8^, respectively. Batch normalization and ReLU activation functions are applied consecutively at the end of each convolutional layer, and the ReLU function is applied at the end of each fully connected layer. After passing the last ReLU function, the data is mapped into one neuron as the output. The number of channels and neurons are hyperparameters that can be tuned for further accuracy. In the current study, we found that a maximum of 10 channels and 4000 neurons leads to sufficient accuracy while at the same time avoiding over‐fitting.

**Figure 2:**
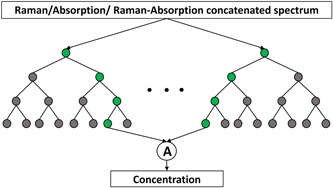
Schematic view of the random forest model composed of multiple decision trees with either Raman, absorption, or concatenated Raman‐absorption spectrum as the input. “A” stands for the average. The number of nodes and trees shown are for illustration purposes and do not reflect the actual values.

RF regression is a supervised machine‐learning technique that utilizes the ensemble average of multiple decision trees to make final predictions (Grömping, 2009). Each one of the trees makes its prediction of the concentration. As shown in Figure 2, the Raman, absorption, or their concatenated spectrum is used as the input with the concentration as the output. RF is a powerful regression technique that runs efficiently on larger data sets. RFs are generally suitable for making predictions in the training range. Additionally, we use the bootstrapping technique, where we select multiple training samples from the original training sample, and these different samples are used for training each one of these decision trees. Bootstrapping reduces over‐fitting chances and stabilizes the network. The squared error criterion in scikit‐learn (Pedregosa et al., 2011) is used to measure the quality of splitting for 100 trees.

## 3 RESULTS AND DISCUSSIONS

We use CNN and RF as two powerful ML techniques, with different levels of preprocessing to identify the optimum predictions. Here, we discuss how the algorithms work with the test data generated using 5‐fold cross‐validation, where each fold can contain points both inside and outside of the training ranges. For CNN, we discuss whether a single or double CNN works better when both Raman and absorption spectra are used as the input.

**Figure 3:**
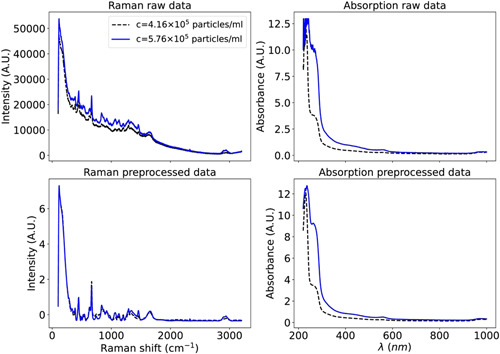
Raw and preprocessed Raman and absorption plots at two different concentrations

In this study, CNN models are composed of multiple convolutional layers with a kernel size of three, where, in each layer, by convolving around the signal, hidden features and patterns are learned. To expedite the learning process and improve the model performance, it is beneficial to preprocess the data before training the models. Thus, we apply baseline corrections and normalize the data using the standard normal variate method, that is, subtracting each spectrum by its mean value and dividing by the standard deviation described by Romero‐Torres et al. (2006) Additionally, normalizing the Raman spectrum makes intensities of the Raman and absorption spectrum to be approximately in the same order for further comparison. No baseline correction or normalization is required for the absorption spectrum since the difference between maximum and minimum values is relatively low. Additionally, normalizing the absorption data led to no significant boost in prediction accuracy. Finally, the Raman and absorption signals are smoothened using the Savitzky‐Golay (SG) filter (González‐Viveros et al., 2021; Romero‐Torres et al., 2006). Figure 3 demonstrates the Raman and absorption spectra before and after preprocessing for two different concentrations. In addition to normalization and applying filters, some studies trim the Raman spectrum to obtain the spectral range of interest (Pian et al., 2022). In the current study, we did not observe any significant gain in the prediction accuracy when the Raman or absorption spectrum is trimmed, as we have shown, for example, for the RF method in the Appendix. Additionally, we analyzed how the predictions change with the subtraction of the control spectrum of solvent as described in the Appendix, where we noticed a reduction in the accuracy with the subtraction of the control spectrum. Therefore, we excluded the subtraction of the control spectrum step from preprocessing steps.

**Figure 4:**
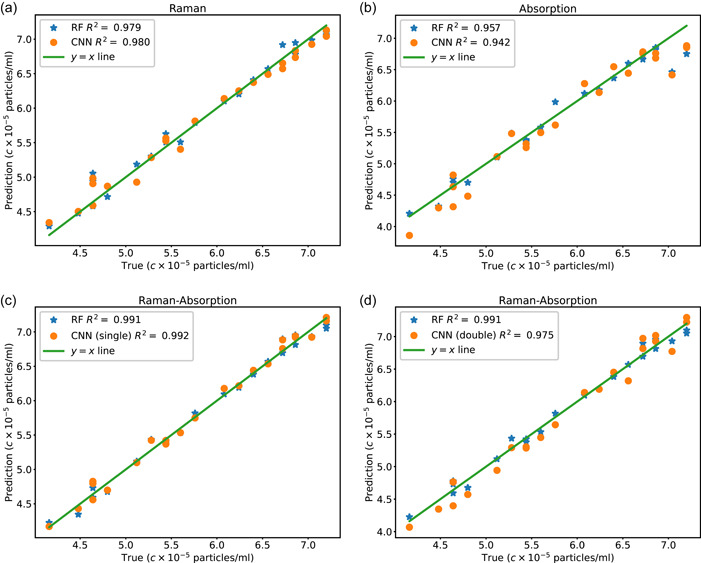
Comparison of the predictions of the RF and CNN for one fold in the 5‐fold cross‐validation datasets when (a) the Raman spectrum (b) the absorption spectrum (c–d) the Raman‐absorption spectrum is used as the input. Cases (c) and (d) correspond to a single and dual network, respectively.

The *R* ^2^ coefficients for the values of the 5‐fold predictions for both RF and CNN are listed in Table 1. The average *R* ^2^ score for all the predictions is above 90%. However, the prediction accuracy is higher when the concatenated Raman‐absorption spectrum is used for RF and CNN compared to the predictions based on either Raman or absorption spectrum. Furthermore, the prediction accuracy is slightly higher for RF compared to CNN in the hyperparameter space we studied. However, both RF and CNN lead to predictions with *R* ^2^ values as high as 98% when the joint Raman‐absorption data is used. Additionally, we note that the single CNN demonstrates higher prediction accuracy compared to the double CNN, which might be attributed to the low predictability of the absorption spectrum compared to the Raman spectrum, as the prediction accuracy is higher when only Raman is used compared to when only absorption data is used. Furthermore, we have made a comparison between RF and support vector machine (SVM) methods in Appendix, where we note that RF predictions are slightly more accurate than SVM. Additionally, we visually demonstrate how the predictions of CNN and RF vary for the testing data set in one of the folds in the 5‐fold data set. As demonstrated in Figure 4, prediction values based on the Raman spectrum are more in line with the actual values as opposed to the absorption spectrum, where the average *R* ^2^ coefficient is lower. This difference can be attributed to the larger size of the Raman signal and, therefore, larger regions of dissimilarity corresponding to different concentrations, which make Raman spectra more distinguishable from each other. Further, the use of joint Raman‐absorption spectra boosts the prediction accuracy compared to the case when only Raman spectra are used.

**Table 1:** The *R* ^2^ values of 5‐fold cross‐validation for the prediction of concentration for given Raman, absorption, and Raman‐absorption concatenated spectra

Fold	Absorption	Raman	Raman‐absorption	Concatenated R‐A	Absorption	Raman	Raman‐absorption
1	0.975	0.976	0.981	0.992	0.977	0.974	0.988
2	0.948	0.973	0.980	0.974	0.974	0.983	0.995
3	0.942	0.980	0.977	0.992	0.964	0.979	0.991
4	0.835	0.964	0.931	0.946	0.881	0.953	0.981
5	0.952	0.984	0.980	0.991	0.989	0.982	0.990
Ave	0.930	0.975	0.969	0.979	0.955	0.974	0.989

**Figure 5:**
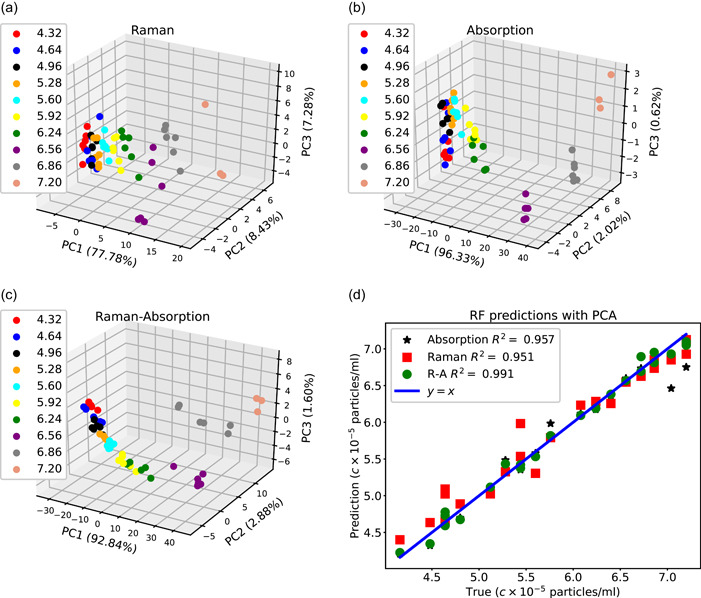
Comparison of the principal component analysis (PCA) plots at different concentration values for (a) the Raman data (b) the absorption data (c) the concatenated Raman‐absorption data. (d) Comparison of RF predictions with dimensionality reduction using PCA for different types of inputs. R‐A stands for the concatenated Raman‐absorption.

The differences between the prediction accuracy of the Raman and the absorption spectra can further be understood through the PCA. We use PCA to reduce the dimensionality of the Raman, absorption, and concatenated Raman‐absorption spectra to 4, where the original size of the Raman and absorption spectra are 3194 and 781. Figure 5 demonstrates how principal coordinate (PC) values differ at different concentration values. The distinction between PCA points at different concentrations is more evident for the Raman‐absorption spectrum as compared to the Raman or absorption spectrum. Additionally, we notice that for most cases, not only the prediction accuracy does not increase by conducting PCA, but also for the Raman and Raman‐absorption data, the average *R* ^2^ values slightly decrease when we compare Figures 4 and 5d. Therefore, for the current data set, dimensionality reduction does not improve the prediction accuracy.

## 4 CONCLUSION

In the current study, the possibility of using absorption, Raman, and joint Raman‐absorption spectrum to determine the concentration of the samples containing viral particles was investigated. RF and CNN, as two different machine learning algorithms, were utilized for making predictions, and the prediction accuracy was monitored using 5‐fold cross‐validation. We demonstrated that with sufficient preprocessing, both the Raman and absorption spectra could be used to create a surrogate to predict the values of concentration. In most cases, the Raman spectrum leads to more accurate predictions compared to the absorption spectrum. Moreover, concatenating Raman and absorption spectra improves the prediction accuracy both for RF and CNN. Furthermore, PCA analysis sheds light on the advantage of joint spectra over single usage of Raman or absorption spectrum as the points corresponding to different concentrations are further separated. We have demonstrated that the joint utilization of the Raman and absorption spectra paves the way for the real‐time measurements of the concentration of the viral particles in well plates, which can be extended to different static and dynamics settings, such as microfluidic devices with different flow conditions.

The key limitations of this study can be listed as follows. (a) the predictions, in general, work well when the unknown concentration values lie in the range of training data sets. Given that here we focused on relatively large concentration values (>4 × 105/ml), the predictions for the low concentration values («4 × 105/ml) are not reliable. (b) The predictions are valid only for ProQuad® samples. Further training data points corresponding to different types of viral particles are required to extend the applicability of the current method.

In future studies, we intend to extend the range of predictions and develop a graphical user interface, which accepts the raw Raman and absorption data as the input and predicts the values of concentrations for different ML methods. Indeed, the current study can serve as a basic block for developing completely automated software that can capture the values of concentration for different types of viral particles using different machine learning algorithms. Furthermore, we aim to extend the predictions to include Raman spectroscopy in microfluidics under different flow conditions.

## AUTHOR CONTRIBUTIONS

**Miad Boodaghidizaji**: Conceptualization (equal); data curation (lead); formal analysis (equal); methodology (lead); software (lead); visualization (lead); writing – original draft (lead); writing – review & editing (lead). **Shreya Milind Athalye**: Conceptualization (equal); data curation (lead); formal analysis (equal); visualization (lead); writing – original draft (lead); writing – review & editing (supporting). **Sukirt Thakur**: Conceptualization (equal); formal analysis (equal); investigation (equal); methodology (lead); software (supporting); writing – original draft (supporting). **Ehsan Esmaili**: Conceptualization (equal); data curation (lead); formal analysis (equal); investigation (equal); writing – original draft (supporting). **Mohit S Verma**: Conceptualization (equal); funding acquisition (supporting); project administration (lead); supervision (lead); writing – review & editing (lead). **Arezoo M Ardekani**: Conceptualization (equal); funding acquisition (lead); project administration (lead); supervision (lead); writing – review & editing (lead).

## CONFLICT OF INTEREST

None declared.

## ETHICS STATEMENT

None required.

## Figures and Tables

**Figure A1:**
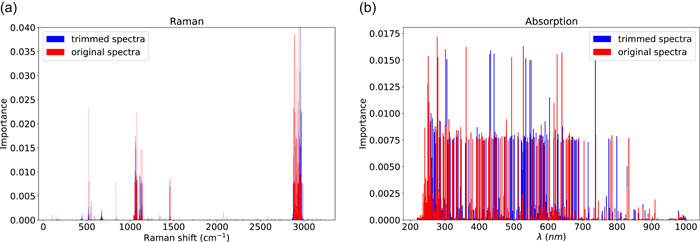
Importance of each point in the (a) Raman shift (b) absorption wavelength in the regression based on the RF method.

**Figure A2:**
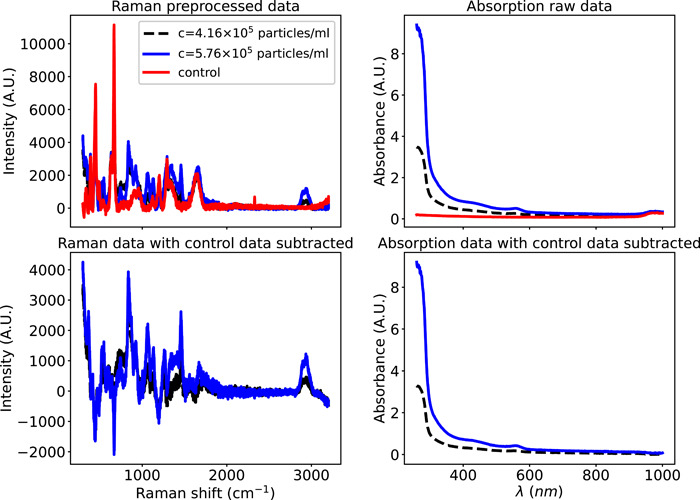
Preprocessed Raman, absorption, and the control (sterile water) spectrum plots in addition to plots with control data subtracted.

## Data Availability

The data sets generated and/or analyzed during the current study are available in the Mendeley data repository at https://doi.org/10.17632/44sgp2jvj5.1.

## 1

Several ML algorithms can be used for making predictions using Raman and absorption spectra. Here, we present how the predictions might be different if one chooses a different ML algorithm, such as the support vector machine (SVM). Table A1 demonstrates the *R* ^2^ coefficients for the values of the 5‐fold predictions for the SVM method. The results are very similar to the values listed for the RF method for the Raman and the concatenated Raman‐absorption spectrum. However, for the absorption spectrum, we note that the RF predictions are more accurate than SVM.

**Table A1:** The *R* ^2^ values of 5‐fold cross‐validation for the prediction of concentration for the Raman, absorption, and concatenated Raman‐absorption spectrum using the SVM method

SVM			
Fold	Absorption	Raman	Raman‐absorption
1	0.664	0.980	0.991
2	0.698	0.984	0.990
3	0.777	0.981	0.989
4	0.601	0.968	0.980
5	0.621	0.984	0.993
Ave	0.672	0.979	0.988

*Note*: Furthermore, we demonstrate how the prediction *R* ^2^ values change if the input dimension is reduced to four using PCA for the RF and SVM methods. As shown in Table A2, we note that dimensionality reduction, in this case, significantly decreases the *R* ^2^ values, particularly for the SVM method.

Abbreviations: PCA, principal component analysis; RF, random forest; SVM, support vector machine.

**Table A2:** The *R* ^2^ values of 5‐fold cross‐validation for the prediction of concentration for the Raman, absorption, and concatenated Raman‐absorption spectrum using PCA

	RF			SVM		
Fold	Absorption	Raman	Raman‐absorption	Absorption	Raman	Raman‐Absorption
1	0.958	0.926	0.933	0.664	0.731	0.701
2	0.972	0.935	0.960	0.698	0.746	0.790
3	0.949	0.951	0.940	0.777	0.623	0.578
4	0.873	0.914	0.973	0.601	0.758	0.793
5	0.973	0.957	0.973	0.621	0.695	0.538
Ave	0.945	0.937	0.956	0.672	0.710	0.680

*Note*: The background noise can affect the Raman and absorption spectra, particularly at low Raman shifts and wavelengths. As a result, in this section, we remove the initial parts of the Raman (Raman shift <300 cm^−^^1^) and absorption spectrum (λ < 250 nm). As shown in Table A3, we note that the prediction accuracies do not change significantly with trimming. Therefore, we used the entire spectra for prediction. Indeed, one of the advantages of using machine learning techniques is that these techniques automatically detect which part of the signal is important. Figure A1 demonstrates the values of importance for the Raman and absorption spectra before and after trimming. The important values are obtained automatically from the Sklearn importance attribute for the RF method (Pedregosa et al., 2011). As evident, we do not notice any significant shift in the important regions of the signals.

Abbreviations: PCA, principal component analysis; RF, random forest; SVM, support vector machine.

**Table A3:** The *R* ^2^ values of 5‐fold cross‐validation for the prediction of concentration for the trimmed Raman, absorption, and concatenated Raman‐absorption spectrum using the RF method

RF			
Fold	Absorption	Raman	Raman‐absorption
1	0.958	0.971	0.987
2	0.972	0.982	0.991
3	0.949	0.967	0.975
4	0.873	0.961	0.955
5	0.973	0.980	0.988
Ave	0.945	0.972	0.979

*Note*: In this study, we did not subtract the control spectrum of the solvent (sterile water) from the Raman and absorption spectrum to minimize the amount of preprocessing. Here, we demonstrate how the prediction accuracies change if we subtract the control data from all the spectrums. Figure A2 demonstrates the comparison of the Raman and absorption spectrum for samples that contain viral particles. As evident, the presence of viral particles induces noticeable changes at most Raman shifts. Further, the absorption signal at all wavelengths is different when viral particles are introduced. Additionally, we presented the spectrum with the water data subtracted. Table A4 demonstrates *R* ^2^ values for predictions of the RF method using the spectrum with water data subtracted. We note that the *R* ^2^ values decrease with the subtraction of water data compared to the values presented in Table A3. Therefore, we excluded the subtraction of the water spectrum step in the preprocessing.

Abbreviation: RF, random forest.

**Table A4:** The *R* ^2^ values of 5‐fold cross‐validation for the prediction of concentration for the trimmed Raman, absorption, and concatenated Raman‐absorption spectrum using the RF method with control data being subtracted

RF			
Fold	Absorption	Raman	Raman‐absorption
1	0.975	0.920	0.981
2	0.982	0.983	0.991
3	0.971	0.931	0.948
4	0.882	0.866	0.917
5	0.989	0.947	0.974
Ave	0.959	0.972	0.962

Abbreviation: RF, random forest.
